# Spontaneous externalization of peritoneal catheter through the abdominal wall in a patient with hydrocephalus: a case report

**DOI:** 10.4076/1757-1626-2-6898

**Published:** 2009-09-16

**Authors:** Theodossios Birbilis, Efthimia Theodoropoulou, Georgios Matis

**Affiliations:** Department of Neurosurgery, University Hospital of Alexandroupolis, Democritus University of Thrace Medical SchoolDragana, GR-68100, AlexandroupolisGreece

## Abstract

Since 1905, the abdominal cavity has been used for absorption of cerebrospinal fluid in patients with hydrocephalus. We report a case of a 33-year-old female, in which a spontaneous extrusion of the peritoneal catheter of a ventriculo-peritoneal shunt through the intact abdominal wall occurred. We suggest that the rather hard peritoneal catheter eroded the abdominal wall, caused local inflammation, and then extruded through the skin. Additionally, the intestinal peristaltic movements, the omental activity and the intraabdominal pressure could play an adjuvant part, pressing direct the foreign body from the peritoneal cavity toward the skin.

## Introduction

The use of peritoneal cavity for cerebrospinal fluid (CSF) absorption in venriculo-peritoneal shunt (VPS) was introduced in 1905 by Kausch, since then VPS is amongst the most frequently performed operations in the management of hydrocephalus [1,2]. Many varied complications related to this procedure have been reported [1-6]. We report a very rare complication of spontaneously externalization of the peritoneal catheter through the intact abdominal wall at an area unrelated to the surgical incision.

## Case presentation

A 33-year-old Greek housewife was admitted to Neurosurgical Department of University Hospital of Alexandroupolis, because of low-grade body temperature (37.9°C) and protrusion of her peritoneal catheter from the abdominal wall. One year earlier, she underwent an installation of a standard-pressure VPS at another institution for obstructive hydrocephalus after a subtotal extirpation of an ependymoma of 4^th^ ventricle.

**Figure 1.**
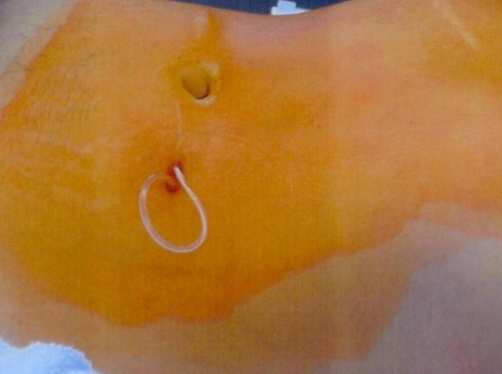
Photograph showing a part of the peritoneal catheter protruding from the abdominal wall.

One week before admission, she developed a localized swelling on the left lower abdominal wall, through which a part of the peritoneal catheter extruded 6 days later (Figure 1).

Physical examinations showed an old scar on the right upper abdomen. The peritoneal catheter protruded from the left abdominal wall near the umbilicus. This area was unrelated to a surgical incision.

**Figure 2.**
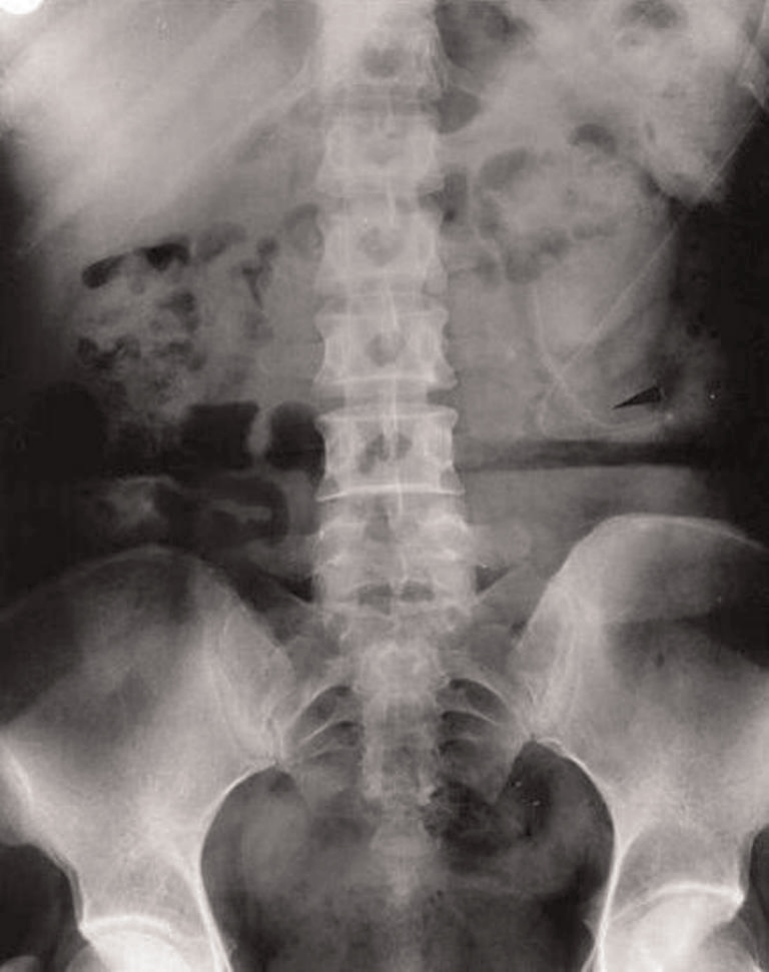
The abdominal X-ray film revealed the course of the peritoneal catheter (arrow).

Laboratory examinations revealed elevations in the white blood cell count (16,200/ml), C-reactive protein levels (8.8 mg/dl), erythrocyte sedimentation rate (89 mm/h). A plain X-ray of the abdomen demonstrated a dislocation of a part of catheter which had partially migrated from the abdominal cavity (Figure 2).

The whole shunting system was immediately removed. The CSF culture demonstrated a *Staphylococcus* epidermis infection and adequate antibiotic treatment was administrated. A new shunting system was installed 6 weeks later after the infection had been controlled. The postoperative course was uneventful.

## Discussion

The peritoneal cavity offers a large surface area of tissue and is the most common site of CSF absorption in hydrocephalus treatment [1,2].

There have been a number of reports of complications relating to the abdominal section of peritoneal shunts [2,3]. Among them, perforation by the peritoneal catheter was reported to have occurred in the vagina, intestine, umbilicus, and the surgical scar of the abdominal wall [1,3].

Several predisposing factors for this complication have been suggested, including infection, multiple shunt revisions, obstruction or dislodgement, peritoneal reaction to stranger body reject, but the pathophysiology is still unknown [1,6].

In our case, we suggest that the rather hard peritoneal catheter eroded the abdominal wall, caused local inflammation, and then extruded through the skin. Additionally, the intestinal peristaltic movements, the omental activity and the intraabdominal pressure could play an adjuvant part, pressing direct the foreign body from the peritoneal cavity toward the skin.

## Abbreviations

CSF: cerebrospinal fluid
VPS: ventriculo-peritoneal shunt
